# Supplementary material for: Snap happy: camera traps are an effective sampling tool when compared with alternative methods
Source: R Soc Open Sci. 2019 Mar 6;6(3):181748. doi: 10.1098/rsos.181748 (PMC6458413; doi:10.1098/rsos.181748)
Supplement: S1 Appendix [file rsos181748supp1.docx]

# Appendix S1 – Definitions of survey methods.

| **Table S1.1.** Definitions of the most common categories of survey methods that were compared to camera traps. | |
| --- | --- |
| Survey method | Definition |
| Plot surveys | Exhaustive searches for animal signs within defined plots |
| Line transects | Searches for animal signs and-or direct sightings of animals along an approximately linear route (such as a path or road) or along a straight transect specifically cut for sampling |
| Live traps | Physical trapping of live animals (e.g. using a cage, box or foothold trap) |
| Hair traps | Passive collection of hair from furred animals, by means of snagging (e.g. using barbed wire or nails) or sticking (e.g. using adhesive tape), and typically involving the use of attractants to encourage animals to approach and interact with the trapping device |
| Track plots | Monitoring of patches of substrate in the environment for animal signs, sometimes involving modification of the substrate (e.g. by adding a layer of sand) |
| Track plates | Monitoring of an artificial surface (the “plate”) inside small trap-like constructions placed in the environment, with ink wells or soot typically used to increase the visibility of tracks |
| Scat surveys | Opportunistic searches for faecal matter, accompanied by DNA sequencing for species identification |
| Detector dogs | The use of specially-trained dogs to locate the scent and-or faeces of target species |
