# Supplementary material for: Snap happy: camera traps are an effective sampling tool when compared with alternative methods
Source: R Soc Open Sci. 2019 Mar 6;6(3):181748. doi: 10.1098/rsos.181748 (PMC6458413; doi:10.1098/rsos.181748)
Supplement: S2 Appendix - Supplementary results [file rsos181748supp2.docx]

# Appendix S2 – Supplementary results.

| **Table S2.1.** Bootstrapped median effect sizes and 95% confidence intervals for each comparison metric. Bootstrapping was not done for metrics with ≤ 15 data points, in which case only the median is presented. | | | |
| --- | --- | --- | --- |
| Metric | *n* | Effect size | 95% confidence interval |
| Accuracy of state variable estimate | 4 | 0.66 | - |
| Cost | 70 | -0.15 | -0.63 - 0.28 |
| Detection probability | 102 | 0.22 | -0.44 - 0.83 |
| Detection rate | 344 | 0.64 | 0.25 - 1.07 |
| Implementation effort | 11 | 0.73 | - |
| Detection latency | 22 | -0.03 | -0.53 - 0.47 |
| Number of individuals detected | 1 | -0.49 | - |
| Precision of state variable estimate | 32 | 0.44 | -0.6 - 1.54 |
| Species richness | 76 | 0.27 | 0.1 - 0.47 |

| **Table S2.2.** Bootstrapped median effect sizes and 95% confidence intervals for each comparison survey method. Bootstrapping was not done for survey methods with ≤ 15 data points, in which case only the median is presented. | | | |
| --- | --- | --- | --- |
| Comparison survey method | *n* | Effect size | 95% confidence interval |
| Acoustic lure | 6 | 4.36 | - |
| Acoustic recorder | 5 | -0.21 | - |
| Active infrared sensor | 1 | -0.91 | - |
| Artificial nest | 4 | -0.46 | - |
| Artificial refuge | 1 | 0.73 | - |
| Continuous video recorder | 2 | 1.37 | - |
| Detector dog | 19 | -0.14 | -1.45 - 0.68 |
| Direct observation | 2 | 0.69 | - |
| eDNA | 3 | 0.40 | - |
| Hair trap | 55 | 1.07 | -0.42 - 2.09 |
| Kill trap | 7 | 1.95 | - |
| Line transect | 158 | 0.23 | -0.39 - 0.94 |
| Live trap | 179 | 0.63 | 0.01 - 1.22 |
| Local ecological knowledge | 6 | 0.04 | - |
| Mist net | 3 | 0.00 | - |
| Photographic survey | 2 | -1.35 | - |
| Playback survey | 1 | -0.49 | - |
| Plot survey | 49 | 0.26 | -0.56 - 1.18 |
| Radio-tracking | 4 | -1.24 | - |
| Scat survey | 35 | 0.19 | -0.57 - 0.93 |
| Track plate | 39 | -0.02 | -0.81 - 0.55 |
| Track plot | 81 | 0.11 | -0.44 - 0.84 |

| **Table S2.3.** Model-averaged parameter estimates (using natural averaging) and 95% confidence intervals for each comparison metric. Parameter estimates are differences from the intercept, with other variables in the models fixed at their baseline factor levels (*Comparison survey method* = Detector dog; *Camera type* = Digital; *Habitat openness* = Closed). Metrics with ≤ 15 data points were not included in models and are excluded from this table. | | |
| --- | --- | --- |
| Metric | Parameter estimate | 95% confidence interval |
| Intercept (Cost) | -0.19 | -1.02 - 0.63 |
| Detection probability | 0.26 | -0.18 - 0.71 |
| Detection rate | 0.71 | 0.34 - 1.08 |
| Detection latency | 0.90 | 0.15 - 1.65 |
| Precision of state variable estimate | 0.79 | 0.13 - 1.46 |
| Species richness | 0.42 | -0.05 - 0.89 |

| **Table S2.4.** Model-averaged parameter estimates (using natural averaging) and 95% confidence intervals for each comparison survey method. Parameter estimates are differences from the intercept, with other variables in the models fixed at their baseline factor levels (*Metric* = Cost; *Camera type* = Digital; *Habitat openness* = Closed). Methods with ≤ 15 data points were not included in models and are excluded from this table. | | |
| --- | --- | --- |
| Comparison survey method | Parameter estimate | 95% confidence interval |
| Intercept (Detector dog) | -0.19 | -1.02 - 0.63 |
| Hair trap | 1.23 | 0.42 - 2.05 |
| Line transect | 0.32 | -0.48 - 1.12 |
| Live trap | 0.43 | -0.37 - 1.23 |
| Plot survey | -0.04 | -0.89 - 0.81 |
| Scat survey | 0.35 | -0.54 - 1.25 |
| Track plate | 0.003 | -0.93 - 0.93 |
| Track plot | -0.02 | -0.86 - 0.82 |
