# Supplementary material for: Snap happy: camera traps are an effective sampling tool when compared with alternative methods
Source: R Soc Open Sci. 2019 Mar 6;6(3):181748. doi: 10.1098/rsos.181748 (PMC6458413; doi:10.1098/rsos.181748)
Supplement: S3 Appendix - Supplementary discussion [file rsos181748supp3.docx]

# Appendix S3 – Supplementary discussion.

# **S3.1. Hair-trapping compared to camera-trapping**

Like camera-trapping, hair-trapping has the benefit of being a minimally-invasive method. However, similar to live-trapping, hair-trapping’s success depends on a series of events which may often fail, in this case persuading an animal to interact with a sticky or sharp surface and deposit hairs, and then successfully identifying the species from the sample (e.g. using DNA analysis). As a result, studies using hair traps often reported few or no detections of species [1–3], even when the traps were optimised for a single species [4–6]. Despite the poor performance of hair traps overall, they remain a valuable survey method because they provide samples which can undergo DNA analysis, which camera traps do not. This allows, for example, individual identification in species without natural markings, and the assessment of population genetic structure (e.g. [7,8]).

# **S3.2. Detector dogs compared to camera-trapping**

The bootstrapped effect sizes indicated that detector dogs were 15% more effective than camera traps (figure 4), based on studies done in the USA [3,9,10] and New Zealand [11]. Although there was no significant difference between the methods on current evidence, further testing of detector dogs as an alternative to camera traps is warranted, focusing on a wider range of habitats and species. Dogs are effective because they can cover a large area, whilst camera traps are effective because they can sample for a long period of time. This difference in the approach means that dogs are likely to be most effective for sampling species with patchy occurrence, whereas camera traps are more effective for species which are periodically unavailable for detection (e.g. species which use burrows or the canopy, or have a home-range extending far beyond the study area). The major drawbacks of using dogs are the high costs associated with the method – though available evidence suggests this is often outweighed by the benefits of higher detection probabilities [3,9,11] – and the limited availability of trained dogs outside the USA (e.g. [6]). Studies using detector dogs are also typically focussed on a relatively small set of species, and camera traps are likely to be more effective for broad-spectrum surveys of whole communities of mammals.

# **S3.3. Metrics for which camera-trapping was least effective**

Camera traps were on average 3% slower at detecting species than other methods and 16% more expensive, though these differences were not significant (figure 4). Given that the camera trap’s forte is sampling continuously for a long period of time, rather than sampling a large area quickly, it is unsurprising that detection latencies were not substantially shorter with camera traps. Neither is it surprising that camera trap surveys were marginally more expensive than other methods, given the considerable initial cost of camera traps. Note, however, that we here quantified the costs of short-term monitoring (typically, a single session of sampling), making our costs a worst-case scenario. Camera traps can be re-used, making them more cost-effective the longer that they are employed [10,12–15].

# **S3.4. Study characteristics which had ambiguous or no relationship with camera trap effectiveness**

Author recommendations were less likely to be in favour of camera traps when attractants were used (figure 3), which was counter to our prediction. One explanation for this is that studies which used attractants with camera traps might have been more likely to compare them with methods which also used attractants (such as live-trapping or track plates), and that these methods may have been highly effective for the objectives of the study and the metrics it used. This explanation is supported by the fact that the mixed-effects modelling of the quantitative data, which controlled for other factors (such as the comparison method and metric used, as well as the random study-to-study variation), did not find an effect of attractants on camera trap effectiveness (table 1). We note also that studies using attractants are unlikely to be a random subset of all camera trap studies in other ways and might, for example, be focussed on species which are unusually rare or otherwise difficult to sample using camera traps.

We found no effect of latitude or minimum species body weight on camera trap effectiveness, for both the author recommendations and the effect sizes (table 1). Although there are theoretical reasons for expecting effects of temperature and body size on camera trap effectiveness, these effects were too small to be detectable across studies. This does not discount the possibility for *within-study* variation in camera trap effectiveness based on these factors, i.e. across camera traps within a study and across species (e.g. see [16]).

# References

1. Paull DJ, Claridge AW, Cunningham RB (2012) Effective detection methods for medium-sized ground-dwelling mammals: a comparison between infrared digital cameras and hair tunnels. Wildl Res 39: 546–553.

2. Swan M, Di Stefano J, Christie F, Steel E, York A (2014) Detecting mammals in heterogeneous landscapes: implications for biodiversity monitoring and management. Biodivers Conserv 23: 343–355.

3. Long RA, Donovan TM, Mackay P, Zielinski WJ, Buzas JS (2007) Comparing scat detection dogs, cameras, and hair snares for surveying carnivores. J Wildl Manage 71: 2018–2025.

4. Comer CE, Symmank ME, Kroll JC (2011) Bobcats do not exhibit rub response despite presence at hair collection stations. Wildl Biol Pract 7: 116–122.

5. Vine SJ, Crowther MS, Lapidge SJ, Dickman CR, Mooney N, et al. (2009) Comparison of methods to detect rare and cryptic species: a case study using the red fox (*Vulpes vulpes*). Wildl Res 36: 436.

6. Riley M, Soutyrina S, Miquelle D, Hayward G, Goodrich J, et al. (2017) Comparison of methods for estimating Amur tiger abundance. Wildlife Biol 1: wlb.00253.

7. Fisher JT, Bradbury S (2014) A multi-method hierarchical modeling approach to quantifying bias in occupancy from noninvasive genetic tagging studies. J Wildl Manage 78: 1087–1095.

8. Velli E, Bologna MA, Silvia C, Ragni B, Randi E (2015) Non-invasive monitoring of the European wildcat (*Felis silvestris silvestris* Schreber, 1777): comparative analysis of three different monitoring techniques and evaluation of their integration. Eur J Wildl Res 61: 657–668.

9. Harrison RL (2006) A comparison of survey methods for detecting bobcats. Wildl Soc Bull 34: 548–552.

10. Clare JDJ, Anderson EM, MacFarland DM, Sloss BL (2015) Comparing the costs and detectability of bobcat using scat-detecting dog and remote camera surveys in central Wisconsin. Wildl Soc Bull 39: 210–217.

11. Glen AS, Anderson D, Veltman CJ, Garvey PM, Nichols M (2016) Wildlife detector dogs and camera traps: a comparison of techniques for detecting feral cats. New Zeal J Zool 43: 127–137.

12. Welbourne DJ, MacGregor C, Paull D, Lindenmayer DB (2015) The effectiveness and cost of camera traps for surveying small reptiles and critical weight range mammals: a comparison with labour-intensive complementary methods. Wildl Res 42: 414–425.

13. Ford AT, Clevenger AP, Bennett A (2009) Comparison of methods of monitoring wildlife crossing-structures on highways. J Wildl Manage 73: 1213–1222.

14. Lyra-Jorge MC, Ciocheti G, Pivello VR, Meirelles ST (2008) Comparing methods for sampling large- and medium-sized mammals: camera traps and track plots. Eur J Wildl Res 54: 739–744.

15. Molyneux J, Pavey CR, James AI, Carthew SM (2017) The efficacy of monitoring techniques for detecting small mammals and reptiles in arid environments. Wildl Res 44: 534–545.

16. Rowcliffe JM, Carbone C, Jansen PA, Kays R, Kranstauber B (2011) Quantifying the sensitivity of camera traps: an adapted distance sampling approach. Methods Ecol Evol 2: 464–476.
